# Fostamatinib treatment for patients with antiphospholipid syndrome and low platelet count: A case series

**DOI:** 10.1111/bjh.70394

**Published:** 2026-02-25

**Authors:** Ekaterina Balaian, Rosa Sonja Alesci, Sandra Marten, Julia‐Annabell Georgi, Christiane Naue, Martin Bornhäuser, Karolin Trautmann‐Grill

**Affiliations:** ^1^ Medical Department I, University Hospital Carl Gustav Carus Dresden TU Dresden Dresden Germany; ^2^ German Cancer Consortium (DKTK) Partner Site Dresden and German Cancer Research Center (DKFZ) Heidelberg Germany; ^3^ IMD Blood Coagulation Center Bad Homburg Germany

**Keywords:** antiphospholipid syndrome, fostamatinib, Syk‐inhibitor, thrombocytopenia

To the Editor,

Antiphospholipid syndrome (APS) is an autoimmune thrombophilic disorder characterized by thromboembolic events and/or pregnancy complications in the presence of persistently positive antiphospholipid antibodies (aPL‐Ab). The standard clinical management includes anticoagulation with vitamin K antagonists or heparin and anti‐platelet therapy.

Thrombocytopenia (TP) is the most common haematological manifestation of APS, occurring in approximately 20%–50% of patients. While severe TP (platelets <50 GPt/L) is rare,^1^ its management remains challenging. Current guidelines lack specific recommendations for treating TP in these patients. Thrombopoietin receptor agonists (TPO‐RAs), standard in immune thrombocytopenia (ITP), must be used cautiously in APS due to the heightened relative prothrombotic risk^2^, ^3^ of 1.82, although not reaching statistical significance in meta‐analysis.^4^ Other treatment options include glucocorticoids; however, their potential side effects limit prolonged use. In patients with life‐threatening bleeding, intravenous immunoglobulins (IVIGs) may be considered.^5^ In difficult‐to‐treat cases, off‐label B‐cell–depleting therapy with rituximab can be used, although long‐term responses are achieved in only 20%–25% of patients.^6^

Fostamatinib, a spleen tyrosine kinase (Syk) inhibitor approved for ITP, has demonstrated low rates of thromboembolic events in clinical trials^7^: in the FIT1^8^ and FIT2^9^ studies, there were no thrombotic events, whereas one deep vein thrombosis (1.7%) has been detected in real‐world data.^10^ Syk inhibition exert anti‐thrombotic effects by blocking SYK‐dependent signalling downstream of immunoreceptor tyrosine‐based activation motif (ITAM)–containing receptors, thereby reducing platelet activation, aggregation and thrombus formation without directly affecting the coagulation cascade.^11^ Inhibition of SYK attenuates collagen‐ and immune complex–mediated platelet activation and thromboinflammatory processes,^12^, ^13^ making fostamatinib a potentially suitable option for TP in APS. However, its use in this patient population has not been systematically studied, which therefore constituted the rationale for the present study.

In patients with ITP, aPL‐Ab can be detected, raising the question of whether TP should be considered a primary condition or secondary to APS. However, distinguishing between the two disorders is not always straightforward, as their clinical features may overlap and their immunomodulatory treatments are often similar.^14^ While ITP is an autoimmune disorder characterized by isolated TP, APS is defined by the presence of persistent aPL‐Ab and thromboembolic events. Importantly, both conditions can present with severe TP and respond to comparable therapeutic approaches, which provides the rationale for evaluating ITP‐directed therapies, such as fostamatinib, in APS‐associated TP.

We report our experience using fostamatinib in nine APS patients with clinically significant TP from two German haemostaseology departments. Data were collected retrospectively by chart review. Informed consent was obtained from all individuals for the use of anonymized clinical data. Patients receiving fostamatinib for ITP who also fulfilled clinical and/or laboratory criteria for APS were eligible for inclusion. A call for such cases was issued within the network of the German ITP Registry and the Working Group on Haemostaseology of the German Society of Hematology and Oncology. Patients were included from 2020 onwards, corresponding to the year of fostamatinib approval in Germany. Patient characteristics are summarized in Table 1.

**TABLE 1:** Baseline demographics and disease characteristics.

#	Sex, age	Duration of ITP prior to fostamatinib (months)	Previous ITP directed therapy	Lowest platelet count prior to start of fostamatinib (GPt/L)	Time between fostamatinib initiation and first clinically relevant response (days)	Duration of fostamatinib therapy (weeks)	Best response to fostamatinib	aPL‐Ab type	Clinical APS criteria	Concomitant autoimmune disorder	Concurrent ITP therapy	Anticoagulation/desaggregation
1	Male, 55 years	82	GCS, TPO‐RA	7		32, ongoing	No response	LA, cardiolipin, β2‐GP	Stroke, arterial occlusion of A. carotis interna	SLE	GCS, cyclophosphamide, HCQ	LMWH → VKA
2	Female, 43 years	233	GCS, TPO‐RA	27	14	125, ongoing	Remission	Cardiolipin, β2‐GP	PE	—	—	LMWH
3	Female, 55 years	1	GCS	23	7	30, ongoing	Complete remission	LA, cardiolipin, β‐GP	Stroke, VTE of V. subclavia and V. axillaris	—	GCS	VKA → LMWH → DOAC
4	Female, 61 years	146	GCS, azathioprine, methotrexate, belimumab, rituximab, leflunomide, IVIG	23	—	9, terminated due to lack of response	No response	LA, cardiolipin, β‐GP	—	SLE	HCQ, GCS, anifrolumab	Aspirin
5	Male, 46 years	306	GCS, ciclosporin, rituximab, azathioprine, belimumab	9	56	28, ongoing	Complete remission	LA, cardiolipin, β2‐GP, phosphatidylserine, annexin V	—	SLE	GCS, HCQ	Aspirin
6	Female, 34 years	195	GCS, TPO‐RA, HCQ	3	28	8, ongoing	Remission	LA, cardiolipin, β2‐GP	Cerebral sinus thrombosis	—	GCS	DOAC
7	Female, 52 years	52	GCS, TPO‐RA	2	4	12, terminated due to hepatic toxicity	Complete remission	LA, cardiolipin, β2‐GP	Renal thrombotic microangiopathy	—	TPO‐RA	VKA → LMWH → DOAC
8	Male, 62 years	17	GCS, TPO‐RA	22	n/a	11	Remission	Cardiolipin	Stroke	—	—	Aspirin
9	Female, 54 years	384	GCS, TPO‐RA	Not available	n/a	65	Complete remission	Annexin V	—	—	GCS	—

*Note*: Response criteria: complete remission—platelets >100 GPt/L (in ≥66% of all measurements); remission—platelets >50 GPt/L (in ≥66% of all measurements).

Abbreviations: aPL‐Ab, antiphospholipid antibodies; APS, antiphospholipid syndrome; DOAC, direct oral anticoagulant; GCS, glucocorticosteroids; HCQ, hydroxychloroquine; ITP, immune thrombocytopenia; IVIG, intravenous immunoglobulins; LA, lupus anticoagulans; LMWH, low molecular weight heparin; n/a, not available; PE, pulmonary embolism; SLE, systemic lupus erythematosus; TPO‐RA, thrombopoietin receptor agonist; VKA, vitamin K antagonist; VTE, venous thromboembolism; β2‐GP, beta‐2‐glycoprotein.

The diagnosis of APS was confirmed according to the revised Sydney criteria, requiring aPL‐Ab positivity within 3 years of an APS‐related clinical event (macro‐ and microvascular arterial and venous thrombosis, obstetric morbidity).^15^ At the time of fostamatinib initiation, five patients (56%) met clinical APS criteria: two had arterial thromboses, one had both arterial and venous thromboembolism, and two had isolated venous thromboembolism. The patient 7 had histologically proven renal thrombotic microangiopathy, diagnosed after fostamatinib had been discontinued due to hepatic side effects with elevation of liver enzymes Common Terminology Criteria for Adverse Events (CTCAE II°). No further side effects of fostamatinib have been detected in all nine patients, specifically no arterial hypertension or gastrointestinal toxicity.

In the remaining three patients, although clinical APS criteria were not met, TP was considered an aPL‐associated manifestation.^1^

All patients received fostamatinib for at least 8 weeks (median 28 weeks, Standard error of the mean (SEM) 12.69, range 8–125). Seven patients (78%) responded to treatment (Figure 1), whereas six of them continued therapy for the entire duration of data collection, while one patient discontinued treatment despite good response due to side effects. Among them, four (57%) achieved complete remission (platelet counts >100 Gpt/L in ≥66% of measurements), while three (43%) achieved partial remission (>50 Gpt/L in ≥66% of measurements). Time to achievement of the first clinically relevant response ranged from 4 to 56 days (median 14 days, SEM 9.49, range 4–56). The first patient has been treated for more than 32 weeks without a clear clinical response. Concomitant therapy was temporarily required in seven of nine patients, including corticosteroids, TPO‐RA, hydroxychloroquine, cyclophosphamide and belimumab, highlighting the importance of bridging therapy during fostamatinib initiation. The precise contribution of concomitant medications to the overall treatment outcome cannot be reliably determined. It cannot be excluded that the observed therapeutic effects were at least partly attributable to the combined use of these agents rather than to the investigational treatment alone.

**FIGURE 1:**
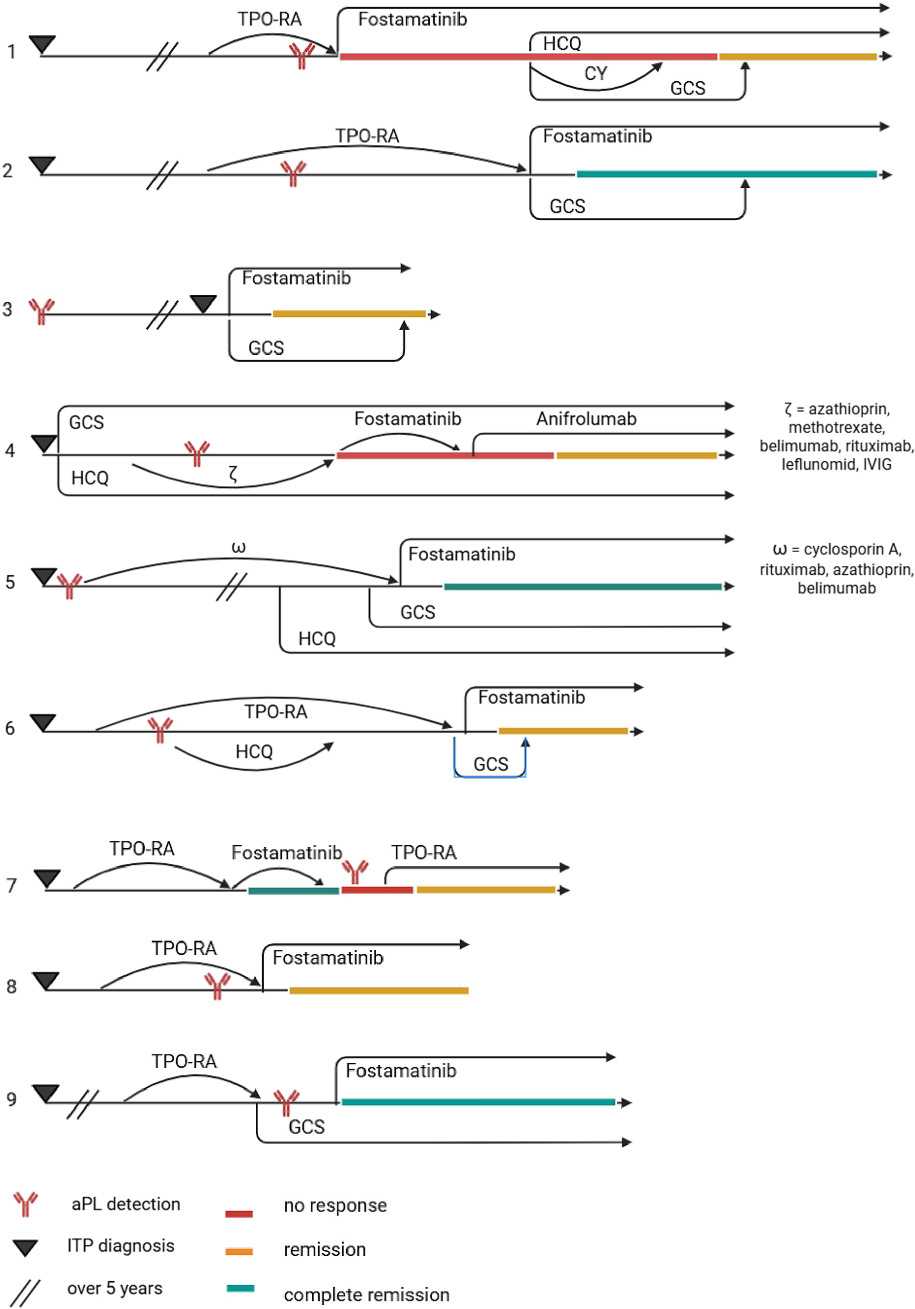
Clinical course of patients treated with fostamatinib. CY, cyclophosphamide (dose not reported); GCS, glucocorticosteroids; HCQ, hydroxychloroquine; IVIG, intravenous immunoglobulins; TPO‐RA, thrombopoietin receptor agonist. Response criteria: complete remission—platelets >100 GPt/L (in ≥66% of all measurements); remission—platelets >50 GPt/L (in ≥66% of all measurements). Lines are illustrative rather than time‐scaled, given the wide disparity in treatment times across patients.

Three patients had systemic lupus erythematosus (SLE) as a relevant comorbidity; two of them were non‐responders to fostamatinib.

Anti‐thrombotic therapy included low‐dose aspirin in patients with arterial thromboembolism or in prophylactic intention, or systemic anticoagulation in cases of venous thrombotic events. In most cases, low molecular weight heparin was preferred due to insufficient platelet counts for the safe use of oral anticoagulants. Three patients were transitioned to oral agents upon platelet stabilization.

In summary, our case series suggests that fostamatinib may be a safe and effective option in APS patients with severe TP. In contrast to other available therapies—such as glucocorticoids, IVIG or rituximab—whose efficacy is often transient or associated with relevant adverse effects, fostamatinib achieved sustained platelet responses without new thromboembolic events during treatment, although this finding is limited by the small patient cohort and the retrospective nature of the analysis. This observation aligns with clinical trial and real‐world data in ITP, where fostamatinib demonstrated low thrombotic risk, and supports its potential suitability for APS patients, in whom prothrombotic complications are a major concern. This therapeutic approach may enable stable platelet recovery, allowing safer implementation of necessary anticoagulation. However, as this conclusion is based on a retrospective case series without a control group, causal relationships cannot be established, and further clinical studies are required to prospectively assess safety and efficacy in this patient population. As such, these findings should be regarded as hypothesis‐generating, highlighting the need for systematic prospective evaluation of fostamatinib in APS‐associated TP.

## AUTHOR CONTRIBUTIONS

EB and KT‐G assembled, analysed, interpreted the data and wrote the paper; EB, RSA, SM, J‐AG, CN, MB and KT‐G contributed clinical data; and all authors approved the final version of the manuscript.

## CONFLICT OF INTEREST STATEMENT

EB received honoraria or is an advisor or consultant for Novartis; received personal funding from the Federal Ministry of Research, Technology and Space, Germany (‘Career Advancement in Multidimensional Tumor Targeting’); and received support for attending meetings and/or travel from AbbVie, Swedish Orphan Biovitrium GmbH and Alexion. RSA has acted as a paid consultant to CSL Behring, Amgen, Bayer, BFSH, Grifols, LFB Pharma, Medlearning, Octapharma, onkowissen, STREAMED UP (Pfizer, Grifols), Sobi, Sanofi, and Takeda Pharma; received funding from Takeda, Sobi, Octapharma, Grifols and Novartis; and meeting/congress support from Takeda, Sobi, Pfizer and Bayer. SM has received personal fees for lectures or consultancy from Bayer, Biotest, Pfizer and Swedish Orphan Biovitrium GmbH. J‐AG served on scientific advisory boards for Jazz Pharmaceuticals and Novartis; received honoraria for lectures from Grifols and Sobi; and received support for attending meetings and/or travel from Amgen. CN received support for attending meetings and/or travel from SOBI and Pfizer. MB served on scientific advisory boards for Jazz Pharmaceuticals; received honoraria for lectures from Astellas, Gilead and Jazz Pharmaceuticals; and received support for attending meetings and/or travel from MSD. KT‐G received honoraria or is an advisor or consultant for Amgen, BMS, Grifols, Novartis, Sanofi, SOBI, Stemline and Takeda.

## Data Availability

Data sharing not applicable to this article as no datasets were generated or analysed during the current study.
